# Comparison of the Neuropathology Induced by Two West Nile Virus Strains

**DOI:** 10.1371/journal.pone.0084473

**Published:** 2013-12-18

**Authors:** Emilie Donadieu, Steeve Lowenski, Jean-Luc Servely, Eve Laloy, Thomas Lilin, Norbert Nowotny, Jennifer Richardson, Stéphan Zientara, Sylvie Lecollinet, Muriel Coulpier

**Affiliations:** 1 Virology (UMR1161), French National Institute for Agricultural Research (INRA), Maisons-Alfort, France; 2 Virology (UMR1161), French Agency for Food, Environmental and Occupational Health and Safety (ANSES), Maisons-Alfort, France; 3 Virology (UMR1161), Paris-Est University, National Veterinary School of Alfort, Maisons-Alfort, France; 4 French National Institute for Agricultural Research (INRA), Nouzilly, France; 5 Histology and Pathological Anatomy, Paris-Est University, National Veterinary School of Alfort, Maisons-Alfort, France; 6 Biomedical Research Center, Paris-Est University, National Veterinary School of Alfort, Maisons-Alfort, France; 7 Institute of Virology, University of Veterinary Medicine, Vienna, Austria; 8 Department of Microbiology and Immunology, College of Medicine and Health Sciences, Sultan Qaboos University, Muscat, Oman; University of Cincinnati School of Medicine, United States of America

## Abstract

Some strains of West Nile virus (WNV) are neuroinvasive and may induce fatal encephalitis/meningitis in a variety of animal species including humans. Whether, however, there is a strain-specific signature in the brain is as yet unknown. Here we investigated the neuropathogenesis induced by two phylogenetically distant WNV strains of lineage 1, WNV_IS98_ and WNV_KUN35 911_. While four-week old C57Bl/6J mice were susceptible to both strains and succumbed rapidly after intraperitoneal inoculation, differences were observed in virulence and clinical disease. WNV_KUN35 911_, the less virulent strain as judged by determination of LD_50_, induced typical signs of encephalitis. Such signs were not observed in WNV_IS98_-infected mice, although they died more rapidly. Histological examination of brain sections also revealed differences, as the level of apoptosis and inflammation was higher in WNV_KUN35 911_- than WNV_IS98_-infected mice. Moreover, staining for cleaved caspase 3 showed that the two WNV strains induced apoptotic death through different molecular mechanisms in one particular brain area. Finally, the two strains showed similar tropism in cortex, striatum, brainstem, and cerebellum but a different one in hippocampus. In summary, our data show that, upon peripheral administration, WNV_IS98_ and WNV_KUN35 911_ strains induce partially distinct lesions and tissue tropism in the brain. They suggest that the virulence of a WNV strain is not necessarily correlated with the severity of apoptotic and inflammatory lesions in the brain.

## Introduction

West Nile virus (WNV) is a member of the *Flaviviridae* family and harbors a positive-sense single-stranded RNA (ssRNA) genome of approximately 11 kb. It is maintained in an enzootic cycle between mosquitoes and birds, but can also infect and cause disease in other vertebrates including humans. First isolated in Uganda in 1937 [1], WNV is now endemic in many parts of the world. It was introduced into North America in 1999, and is now the leading cause of mosquito-borne and epidemic encephalitis in the United States with more than 200 human fatalities annually (CDC, http://www.cdc.gov/ncidod/dvbid/westnile/index.htm). In Europe, human infections have been reported for over 50 years [2,3], with an increasing number of outbreaks in recent years [4]. In most cases, the infection remains asymptomatic. In 20% of cases, however, patients develop a mild flu-like illness [5], while severe neuroinvasive disease such as encephalitis, meningitis or acute flaccid paralysis, all of which may be fatal or accompanied by long term neurological sequelae [6], occurs in less than 1% of cases [7]. 

Two major lineages are widely recognized, lineage 1, which includes isolates from Africa, Europe, the Middle East, India, America and Australia (formerly Kunjin virus), and lineage 2, which, while restricted to sub-Saharan Africa and Madagascar prior to 2004, is now widespread in central [8–11], southern [12] and eastern [13] Europe. *In vivo* experiments in mice have indicated that virulence is unrelated to the virus lineage but is strain-specific and dependent on the capacity of the strain to invade the central nervous system (CNS). When inoculated intracerebrally, all strains are indeed virulent and induce encephalitis, whereas few of them are neurovirulent when inoculated at the periphery [14–17]. Once in the CNS, WNV infects neurons [18] and induces lesions characterized histologically by meningitis, perivascular infiltrates, microglial nodules, astrogliosis and neuronal loss. Several brain regions, including cortex, hippocampus, cerebellum, brainstem and anterior horn of the spinal cord [19,20] sustain damage. The mechanisms responsible for neuronal death are currently under investigation. They have been shown to involve both caspase 3-dependent and -independent apoptotic pathways, but the relative contribution made by viral cytopathogenicity and inflammation to cell demise remains to be elucidated (for review, see 21).

It is as yet unknown whether all WNV strains, after entering the brain parenchyma, damage the brain in a similar manner, or whether lesions are strain-specific. Here we investigated the neuropathogenesis induced by two phylogenetically distant WNV strains of lineage 1, WNV_IS98_ and WNV_KUN35911_. WNV_IS98_ is a highly virulent clade 1a strain isolated from a stork with severe neurological symptoms during the 1998 epidemic in Israel [22], and WNV_KUN35 911_, a clade 1b strain originating from a sick horse in 1984 in New South Wales, Australia [23]. While both strains were neuroinvasive in 4-week old C57Bl/6J mice, our results revealed certain differences in clinical disease, apoptotic and inflammatory brain lesions and neurotropism. Unexpectedly, the less virulent strain induced greater damage in several brain areas, suggesting that the virulence of a WNV strain is not directly linked to the intensity of apoptosis and inflammation in the brain parenchyma. 

## Results

In the present study, the virulence and neuropathogenicity of two lineage 1 WNV strains, WNV_KUN35911_ and WNV_IS98_, were comparatively evaluated in 4-week-old C57BL/6J mice. The genetic characterization of both virus strains corroborated previously published data [23,24]. Indeed, the complete genomic sequence of our WNV_IS98_ strain was identical to that reported by Lucas et al., 2004 [24]. Also, partial sequencing of the NS5 gene of the WNV_KUN35911_ strain confirmed its classification in clade 1b (Figure S1A). And finally, the deduced amino acid sequence of E and NS5 genes demonstrated either the presence (in WNV_IS98_) or absence (in WNV_KUN35911_) of 2 virulence markers, namely, the N-glycosylation site in the viral envelope and the phenylalanine residue at position 653 in the NS5 protein (Figure S1B-C).

### Virulence of WNV_KUN35911_ and WNV_IS98_ in 4-week-old C57Bl/6J mice

The virulence of the WNV_KUN35911_ strain had not been previously evaluated in mice. As expected, WNV_IS98_ proved to be more virulent than WNV_KUN35911_ (LD_50_ values of 1.5 and 17 PFU, respectively) (Table 1), although the virulence of WNV_KUN35911_ was much higher in our murine model than usually observed for WNV_KUN_ strains. Of note, clinical assessment of diseased mice revealed differences between groups inoculated with the two strains. The mean survival time was dependent on the inoculated infectious dose in WNV_KUN35911_-infected mice (values ranged from 6.5 to 13 days p.i.), but not in WNV_IS98_-infected mice (values ranged from 7.2 to 7.8 days p.i.) (Table 2). While both strains induced weight loss before death (Table 2), only WNV_KUN35911_-infected mice showed typical signs of encephalitis such as ruffled fur, hunched posture and, ultimately, hind leg paralysis (data not shown). Such symptoms were never observed in WNV_IS98_-infected mice, suggesting that WNV-induced CNS lesions were strain-specific. This prompted us to compare the lesions that the two WNV strains induced in the brain. To this end, brains of mice inoculated with similar infectious doses (10^2^ PFU of each strain) and similar LD_50_ (100 LD_50_, corresponding to 10^2^ and 10^3^ PFU for WNV_IS98_ and WNV_KUN35911_, respectively) were further analyzed. 

**Table 1:** Virulence of WNV_IS98_ and WNV_KUN35911_ in 4-week-old C57BL/6J mice.

Doses (PFU)	Mortality (dead/total)	
	IS98	KUN35911
0.01	0/5	nd
0.1	0/5	1/5
1	3/5	2/5
10	5/5	2/5
10^2^	5/5	4/5
10^3^	nd	5/5
10^4^	nd	4/5
10^5^	nd	5/5
LD_50_ (PFU)^a^	1.5	17

nd: not determined.

^a^ LD50 values were calculated using the Reed and Muench method

**Table 2:** Clinical illness in 4-week-old C57BL/6J mice infected with WNV_IS98_ and WNVKUN_35911_

Doses (PFU)	Onset of weight loss (dpi)		Mean survival time (dpi)	
	IS98	KUN35911	IS98	KUN35911
0.01	na (5/5)	nd	na	nd
0.1	na (5/5)	13.0 ± 0.0 (1/5)	na	13.0 ± 0.0
1	6.0 ± 0.0 (3/5)	9.0 ± 1.2 (5/5)	7.3 ± 0.4	10.0 ± 0.0
10	5.4 ± 0.5 (3/5)	6.3 ± 0.9 (3/5)	7.2 ± 0.3	8.5 ± 1.5
10^2^	6.0 ± 0.8 (5/5)^1a, b^	6.5 ± 0.8 (4/5) ^1a^	7.8 ± 0.6 ^2a, b^	9.5 ± 1.5 ^2a^
10^3^	nd	6.0 ± 0.0 (5/5) ^1b^	nd	8.4 ± 0.5 ^2b^
10^4^	nd	5.6 ± 0.6 (5/5)	nd	6.5 ± 0.5
10^5^	nd	6.0 ± 0.0 (5/5)	nd	7.8 ± 0.3

Values show the mean number of days post-inoculation (dpi) ± SD. The number of symptomatic mice is indicated in parentheses. Statistical analyses between groups (a, b) were made with Wilcoxon test, 1a, 1b, 2b: ns, not significant, *, 2a: p<0.1. nd: not determined, na: not applicable due to the absence of weight loss/mortality.

### WNV_KUN35911_ and WNV_IS98_ induce distinct patterns of apoptotic death in the brain

**Figure 1:**
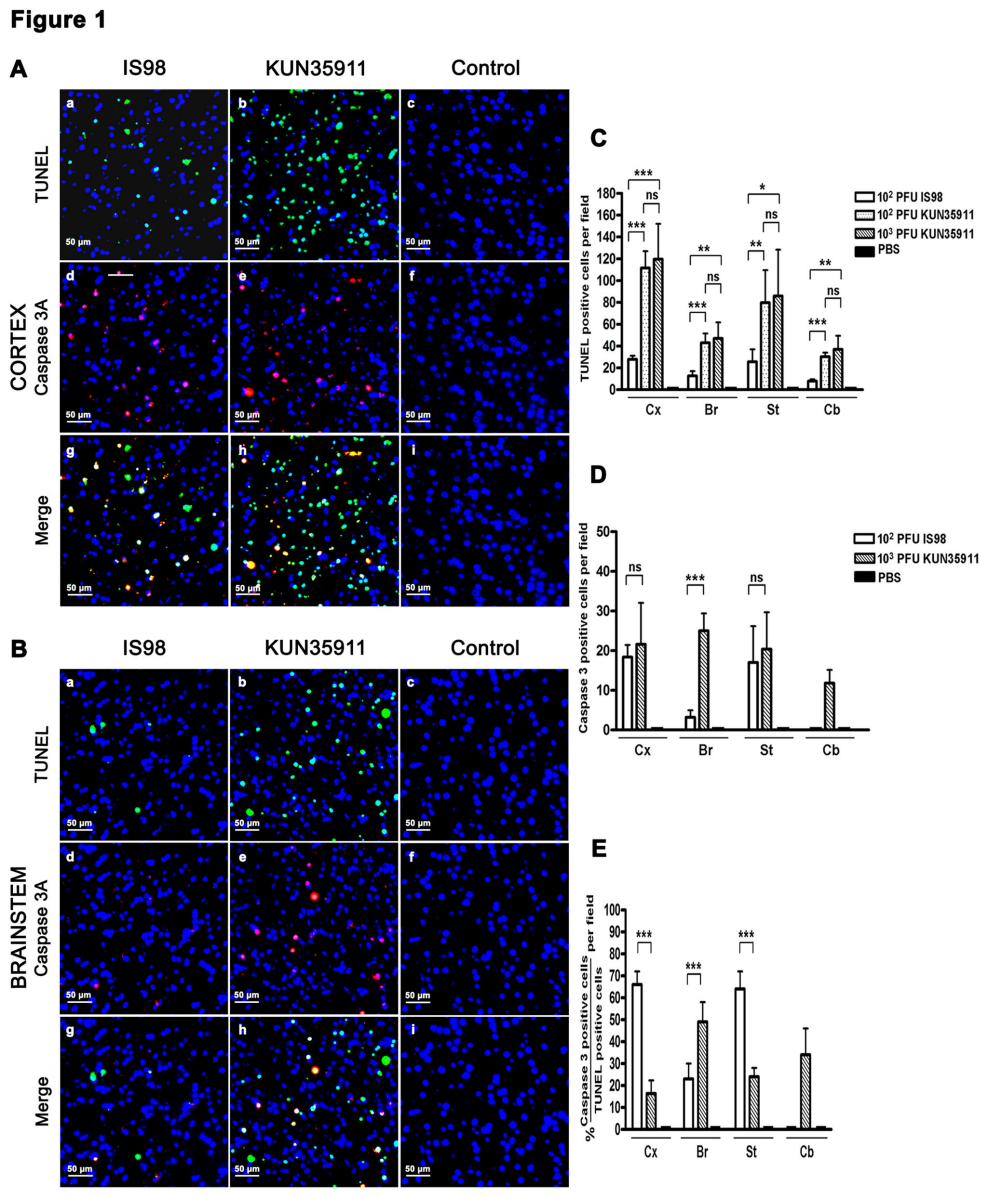
Comparison of WNV_IS98_- and WNV_KUN35911_-induced apoptosis in cortex, brainstem, striatum and cerebellum. Photomicrographs are representative of (A) cortex and (B) brainstem. Four micrometer coronal brain sections were stained for TUNEL (a-c, green), cleaved caspase 3 (d-f, red) and merged (g-i). Total number of (C) TUNEL-positive cells and (D) cleaved caspase 3-positive cells in brain areas. (E) Percentage of cleaved caspase-3 positive cells among TUNEL-positive cells. Two sections were counted per mouse, two fields per section. Data represent the mean values +/- SD from 4 (WNV_KUN35911_ – 10^2^ PFU) to 5 (WNV_KUN35911_ – 10^3^ PFU and WNV_IS98_ – 10^2^ PFU) mice. Statistical analyses were performed by employing the One-way ANOVA test. ns, not significant, *, P<0.1, **, P<0.01, ***, P<0.001. Cx: cortex, Br: brainstem, St: striatum, Cb: cerebellum.

**Figure 2:**
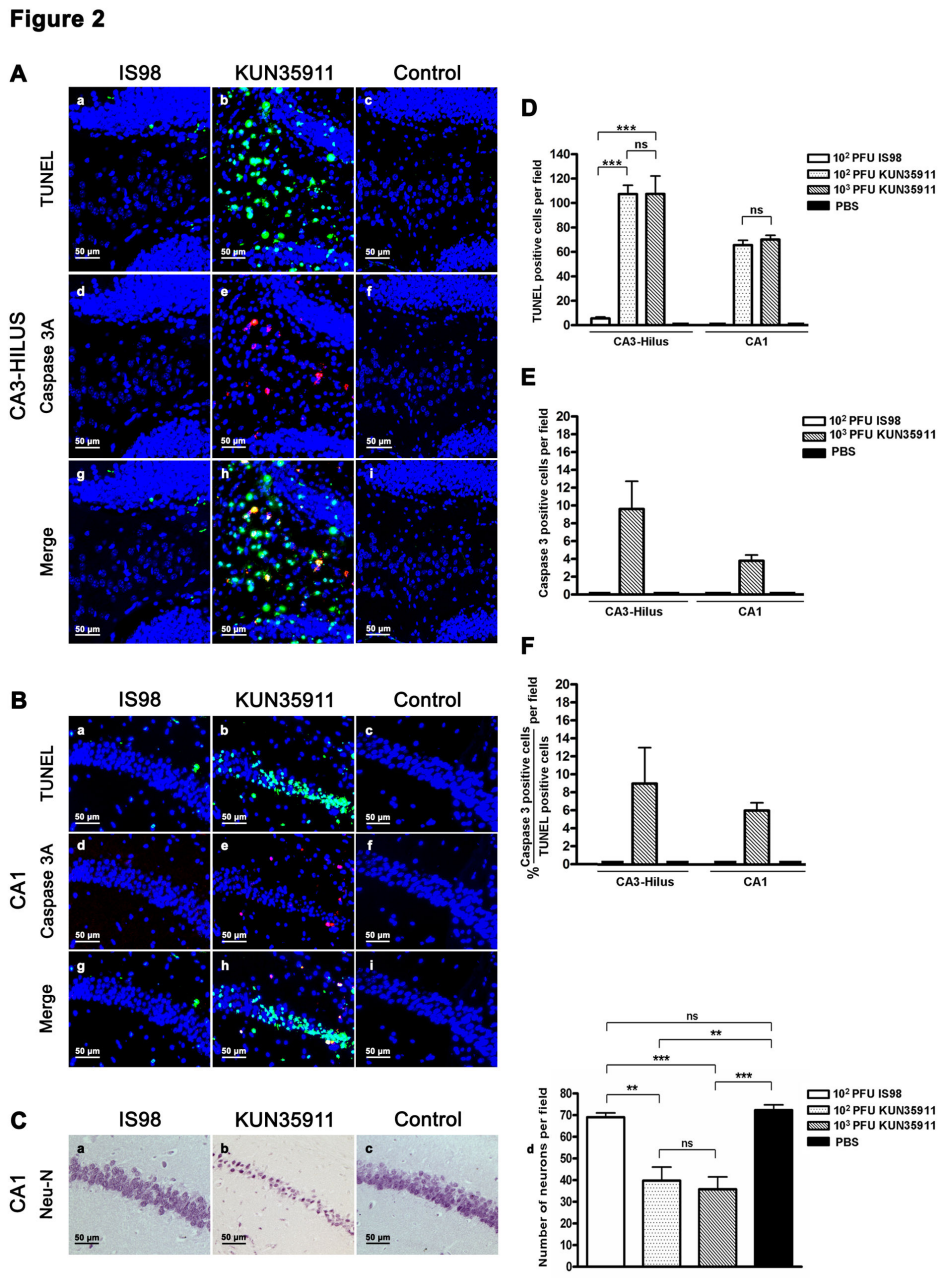
Comparison of WNV_IS98_ and WNV_KUN35911_-induced apoptosis in hippocampus. Photomicrographs are representative of (A) CA3-hilus and (B) CA1 areas. Four micrometer coronal brain sections were stained for TUNEL (a-c, green), cleaved caspase 3 (d-f, red) and merged (g-i). (C) Neuronal loss in the CA1 pyramidal layer of WNV_KUN35911_- but not WNV_IS98_-infected mice. Brain sections were immuno-stained with anti-NeuN (a-c). (d) Total number of neurons per field in the CA1 area. Total number of (D) TUNEL-positive cells and (E) cleaved caspase 3-positive cells. (F) Percentage of cleaved caspase-3 positive cells among TUNEL-positive cells. Two sections were counted per mouse. Data represent the mean values +/- SD from 4 (WNV_KUN35911_ – 10^2^ PFU) to 5 (WNV_KUN35911_ – 10^3^ PFU and WNV_IS98_ – 10^2^ PFU) mice. Statistical analyses were performed by employing the One-way ANOVA test. ns, not significant, **, P<0.01, ***, P<0.001.

In order to study the damage induced by the two WNV strains in the brain, we examined apoptosis, a hallmark of WNV infection that has been described in several brain areas, including cortex, striatum, hippocampus, brainstem and cerebellum. The brains of 4 to 5 mice infected by the intraperitoneal route with 10^2^ PFU of WNV_IS98_ (n=5) or 10^2^ (n=4) and 10^3^ (n=5) PFU of WNV_KUN35911_ were analyzed at terminal stage of the disease. TUNEL staining revealed apoptotic features in almost all areas of the brain in infected mice, regardless of the WNV strain. However, while apoptotic cells were rare in cortex, striatum, brainstem and cerebellum of mice infected with the WNV_IS98_ strain, they were much more numerous in mice infected with the WNV_KUN35911_ strain (Figure 1Aa-c, 1Ba-c, not shown in striatum and cerebellum). Enumeration of TUNEL-positive cells confirmed this observation (Figure 1C). Similar observations were made in hippocampus (Figure 2Aa-c, 2Ba-c). In mice infected with the WNV_KUN3591_ strain, two areas were particularly damaged, the CA3-hilus (Figure 2Ab) and the CA1 (Figure 2Bb) areas of the pyramidal layer. In both regions, TUNEL staining was exclusively (CA1) or almost exclusively (CA3-hilus) restricted to WNV_KUN35911_-infected mice, which was confirmed by enumeration (Figure 2D). Apoptotic staining in the CA1 area was correlated with a strong reduction in the pyramidal layer, as shown in sections immuno-stained with anti-NeuN, an antibody specific for neuronal cells, whereas no reduction was observed in the same area of WNV_IS98_-infected mice, as compared with controls (Figure 2Ca-c). Again, enumeration of surviving neurons confirmed the histological observation (Figure 2Cd). Fifty to 55% of pyramidal neurons were lost in the CA1 area of mice inoculated with either 10^2^ or 10^3^ PFU of WNV_KUN35911_, whereas no loss was evidenced in the same area of WNV_IS98_-infected mice. In an attempt to determine whether TUNEL staining at terminal stage gave a true picture of the dynamic loss of neurons which might occur over time in other brain areas, we performed TUNEL staining in mice infected for 6 days. Although the virus had entered the brain at this time (our own observation), positively stained cells were very rare in WNV-infected mice regardless of WNV strain (data not shown). This suggested that apoptotic death was an event occurring late in the course of the disease. 

In order to gain insight into the molecular mechanisms involved in WNV_KUN35911_- and WNV_IS98_-induced apoptosis, sections were co-stained with TUNEL and an antibody directed against cleaved caspase 3, a protein of the caspase family that participates in the induction and execution phase of apoptosis. As previously shown in a model of WNV_NY99_-induced apoptosis [25], cleaved caspase 3 staining was observed in all brain areas with positive TUNEL staining (Figure 1Ad-i, 1Bd-i, 2Ad-i, 2Bd-i), confirming occurrence of a caspase 3-dependent mechanism of neuronal death. That was confirmed by enumeration of cleaved-caspase 3 positive cells (Figures 1D, 2E). In the brain of WNV_IS98_-infected mice, however, the percentage of TUNEL-positive cells that expressed cleaved caspase 3 (Figure 1E and Figure 2F) was high in cortex and striatum but low in brainstem. Thus, the predominant mechanism of apoptotic death differed in distinct brain structures, being predominantly caspase 3-dependent in cortex and striatum, but independent in brainstem. We were unable to determine a preferred pathway in cerebellum and hippocampus, as the level of WNV_IS98_-induced apoptosis was too low. By contrast, upon infection of mice with the WNV_KUN35911_ strain, the percentage of cleaved caspase 3-positive cells was low (below 50%) in almost all areas of the brain (Figure 1E and Figure 2F), suggesting that a caspase 3-independent mechanism was mainly responsible for WNV_KUN35911_-induced apoptosis. Our results thus demonstrate that the two WNV strains induce distinct pathways of apoptotic death in particular brain areas, and notably in cortex and striatum. 

**Figure 3:**
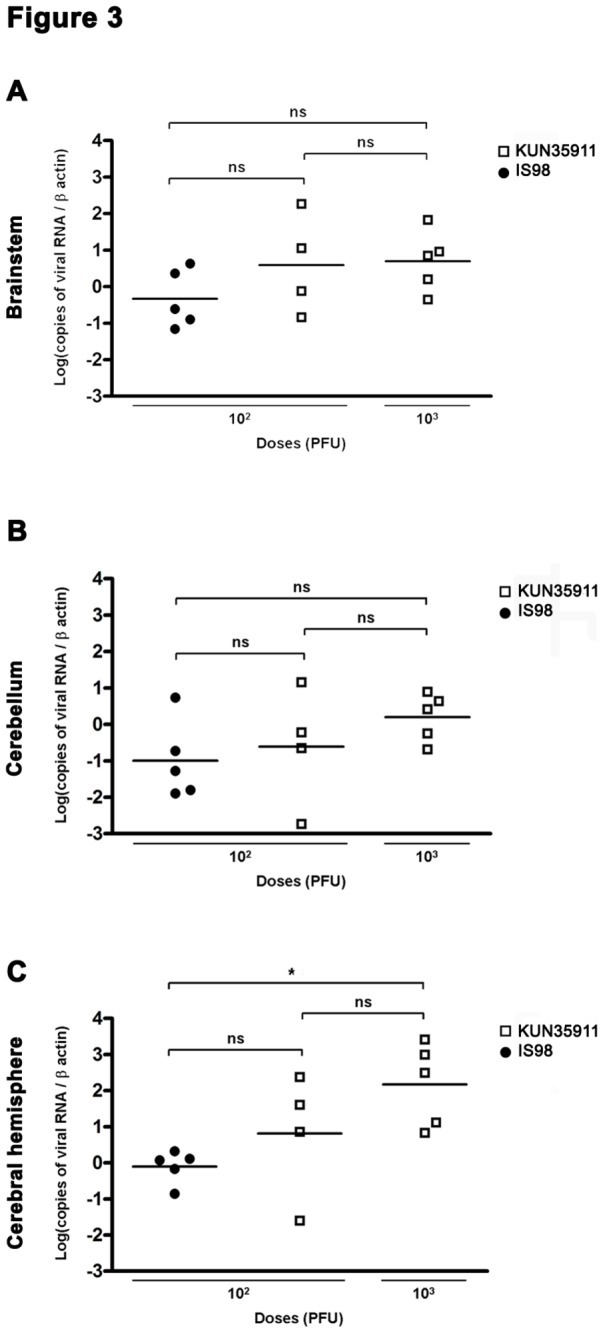
Viral load in brainstem, cerebellum and cerebral hemisphere of WNV_IS98_- and WNV_KUN35911_-infected mice. Absolute copy numbers of viral genomes were quantified by real-time RT-PCR and normalized to β-actin in (A) brainstem, (B) cerebellum and (C) cerebral hemisphere. Each data point represents one animal. The data are the mean values from 4 (WNV_KUN35911_ – 10^2^ PFU) to 5 (WNV_KUN35911_ – 10^3^ PFU and WNV_IS98_ – 10^2^ PFU) mice. Statistical analysis was performed by employing the One-way ANOVA test. ns, not significant, * , P<0.05.

Since a higher level of apoptosis may simply be the consequence of a higher viral load in the brain, we quantified the genomic viral RNA in cerebral hemisphere, brainstem and cerebellum, using quantitative real time RT- PCR (Figure 3). No viral RNA was detected in the brain of control mice inoculated with PBS. In brainstem and cerebellum, viral loads did not differ in a statistically significant manner between the two strains at any of the doses used (Figure 3A, B). Similarly, a statistically significant difference was not observed in the cerebral hemisphere of mice inoculated with 10^2^ PFU of each strain. A significant increase, however, was observed in this structure in mice that received a higher dose (10^3^ PFU) of WNV_KUN35911_ (Figure 3C). Nevertheless, no difference was evidenced in the level of apoptosis induced by 10^2^ or 10^3^ PFU of WNV_KUN35911_ in any brain area studied (Figure 1C and Figure 2D), indicating that the difference observed in viral load had no impact on the level of neuronal apoptosis. Thus, WNV_KUN35911_ induces more severe apoptosis than WNV_IS98_ for similar viral load.

### Neurotropism of WNV_KUN35911_ and WNV_IS98_ strains

**Figure 4:**
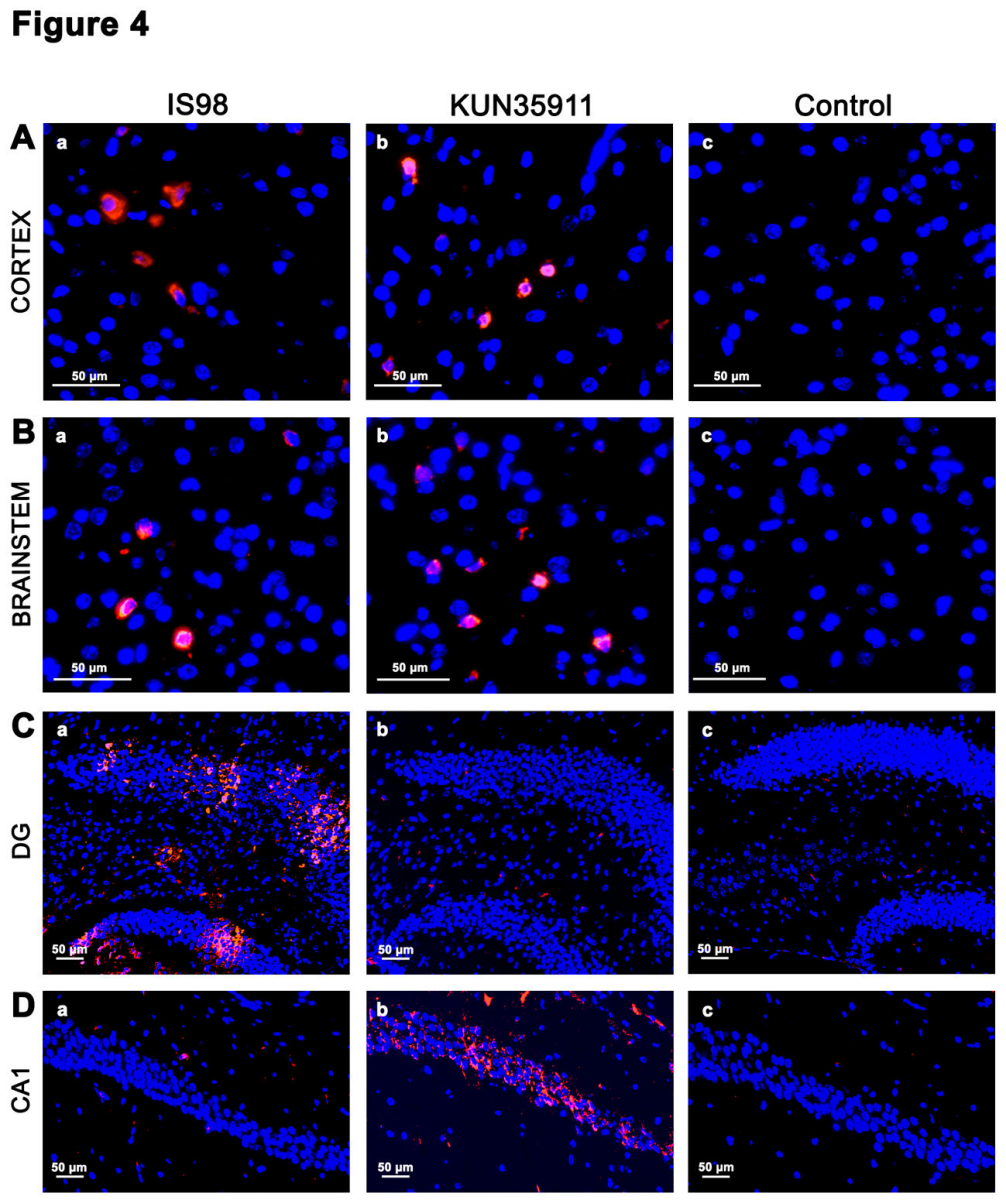
Neurotropism of WNV_IS98_ and WNV_KUN35911_ in mice brains. Coronal brain sections were immuno-stained with E24, an antibody directed against WNV envelope. Photomicrographs are representatives of (A) cortex, (B) brainstem (C) dentate gyrus (DG) and (D) CA1 pyramidal layer. Note the similar patchy distribution of the 2 strains in cortex and brainstem and their differential tropism in DG and CA1 of hippocampus.

WNV-induced neuronal apoptosis is thought to be caused either by a direct cytopathic effect of the virus or by pathological consequences of inflammation (for review, see 21). In order to determine whether the difference observed in the extent of apoptosis induced by the 2 strains was attributable to differential tropism for neural tissue, we analysed the localization of the virus in the brain. Sections were immuno-stained with E-24, an antibody directed against the viral envelope (Figure 4). No staining was observed in control mice that had been inoculated with PBS. In four areas of the brain, in which WNV_KUN35911_ induced a higher level of apoptosis than WNV_IS98_ (cortex, striatum, brainstem, and cerebellum), the distribution pattern of WNV antigen was patchy (Figure 4Aa-c, 4Ba-c and data not shown). Major differences were observed neither in localization nor in intensity of staining, showing that within the parenchyma the two strains of WNV exhibited similar tropism. By contrast, a clear difference in tropism was observed in the hippocampus, as shown in Figure 4 C and D. WNV antigen was mainly present in the CA1 area in WNV_KUN35911_-infected mice (Figure 4D) or in granular cells of the dentate gyrus in WNV_IS98_-infected mice (Figure 4C). Thus, WNV_KUN35911_ infection gave rise to intense antigen expression and apoptosis (Figure 2Bb) in the CA1 area, suggesting that apoptotic death in this area may be due to a direct cytopathic effect of the virus. In contrast, strong WNV_IS98_ antigen expression in dentate gyrus was not accompanied by apoptosis (not shown), indicating that these neurons are insensitive to WNV_IS98_. Thus, the two viral strains displayed differential neurotropism, and more particularly, non-overlapping viral antigen expression in the CA1 and dendate gyrus of the hippocampus. Moreover, while expression of WNV _KUN35911_ antigen in the CA1 was associated with neuronal apoptosis, expression of WNV_IS98_ antigen in the dendate gyrus was not.

### WNV_KUN35911_ and WNV_IS98_ induce distinct inflammatory lesions in the brain

**Figure 5:**
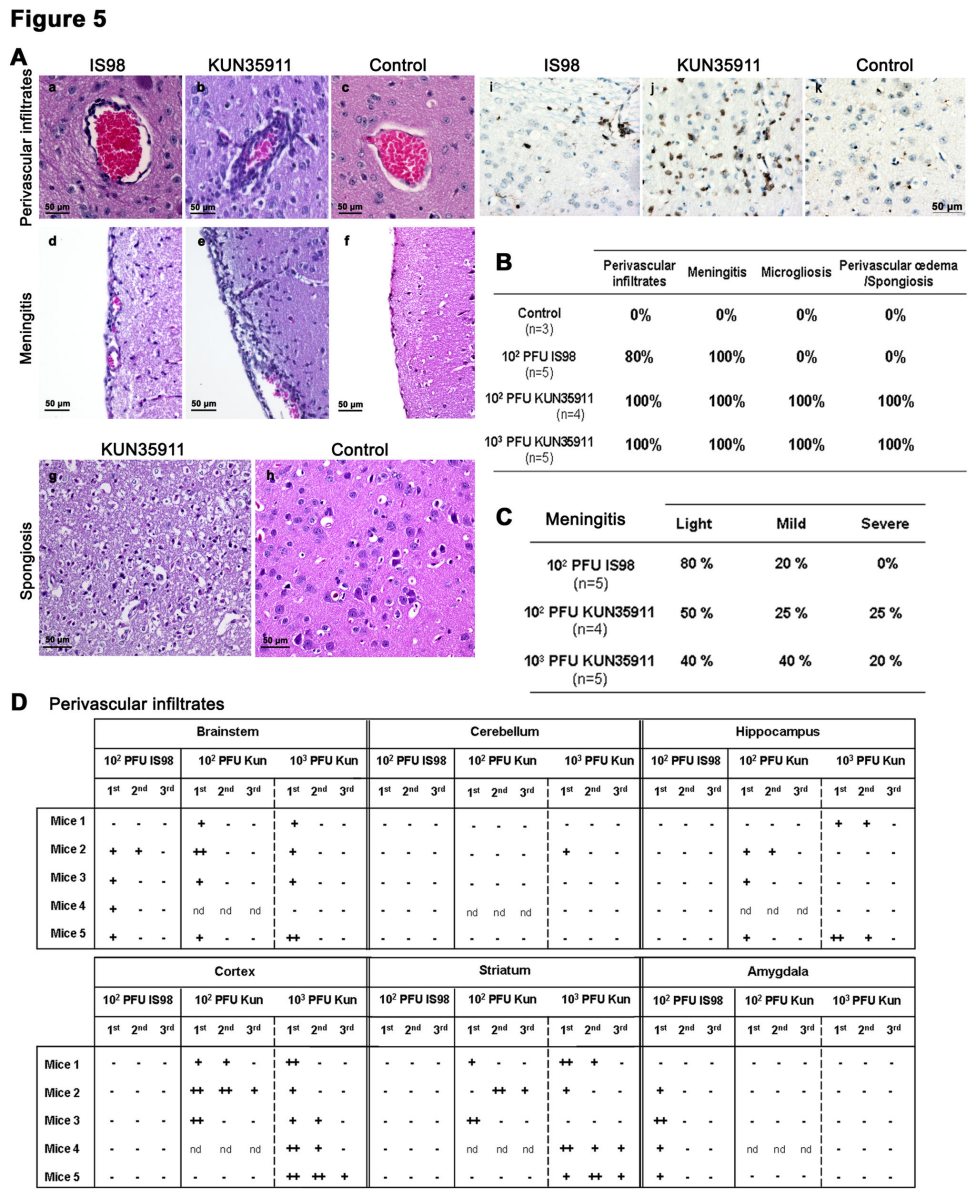
Comparison of inflammatory lesions in the brain of WNV_IS98_- and WNV_KUN35911_-infected mice. (Aa-h) Representative images showing perivascular infiltrates, meningitis and spongiosis in the brain of WNV-infected mice and their controls. Four micrometer coronal sections were stained with hematoxylin-eosin-saffron (HES). Note that third category perivascular infiltrates (b), severe meningitis (e) and spongiosis (g) were observed only in WNV_KUN35911_-infected mice, while first category perivascular infiltrates (a) and mild meningitis (d) were the most frequent in mice infected with WNV_IS98_. No lesions were observed in control mice (c, f and h). (Ai-k) Coronal brain sections were immuno-stained with anti-CD3, an antibody directed against T lymphocyte. Photomicrographs are representative of cortical parenchyma in WNV_IS98_- (i), WNV_KUN35911_- (j) and non- (k) infected mice. (B) Percentage of mice showing signs of inflammation such as perivascular infiltrates, meningitis, microgliosis and perivascular œdema/spongiosis in the brain. (C) Percentage of mice showing mild, moderate or severe meningitis in each group. In (B) and (C) the number of animals in each group is given in parentheses. (D) Quantification of perivascular infiltrates in several brain structures. First, 2^nd^ and 3^rd^ categories were defined by the number of rows of macrophages and lymphocytes surrounding the vessels, respectively 1, 2 and 3 rows. - = no infiltrate, + = 1 to 3 infiltrates, ++ = 4 to 10 infiltrates per section, nd = not determined.

As neuroinflammation is known to contribute to WNV-induced apoptosis [26], we hypothesized that at least part of the difference in the level of apoptosis induced by the two viral strains could be explained by their differential capacity to trigger inflammation in the brain. In order to elucidate this point, sections were stained first with HES, and analyzed for the presence of perivascular infiltrates, meningitis, microgliosis and spongiosis, 4 markers of neuroinflammation (Figure 5) and second with an antibody directed against CD3, a marker of T lymphocytes. Initial examination of HES sections allowed determination of the percentage of animals in each group that showed signs of inflammation (Figure 5B). While lesions were not observed in control PBS-inoculated mice, almost all of the WNV-infected mice had perivascular infiltrates and meningitis. Certain lesions, however, including spongiosis which corresponds to the vacuolization of the neuropil and focal proliferation of microglial cells (microglial nodules), mostly surrounding degenerative neurons, were only observed in WNV_KUN35911_-infected brains (Figure 5Ag-h, 5B). These latter finding was correlated with the paucity of apoptotic cells previously described in the brain of WNV_IS98_-infected mice (Figures 1 and 2). A closer examination of sections showed that 50 to 60 % of WNV_KUN35911_-infected mice but not more than 20 % of those infected by WNV_IS98_ had moderate to severe meningitis (Figure 5C). Severe meningitis was in fact restricted to WNV_KUN35911_-infected brains, whereas infection with WNV_IS98_ led at most to moderate meningitis, as shown in Figure 5 Ad-f. Precise scoring of perivascular infiltrates was also informative (Figure 5D). Three categories (1 to 3), based on the number of rows of immune cells surrounding the vessels, were defined. In mice infected with WNV_IS98_, perivascular infiltrates were not observed in most of the brain areas examined, including cerebellum, hippocampus, cortex and striatum. While infiltrates were, however, consistently observed in brainstem and amygdala these were mostly of grade 1 (Figure 5Aa). By contrast, in mice upon infection with the WNV_KUN35911_ strain, perivascular infiltrates were numerous and more severe in cortex and striatum, with scores as high as grade 3 (Figure 5Ab). No major differences, however, were observed between the two strains in brainstem and cerebellum, showing once again that the differential pathological impact of the two strains is confined to particular areas of the brain. Further examining the entry of inflammatory cells in the brain, we found that some vessels were clearly surrounded with small rounded T cells in the brains of infected mice whereas only very rare T cells were observed in non-infected mice, as shown by anti-CD3 immunostaining. In addition, the frequency of T cells appeared higher in cortical parenchyma (Figure 5Ai, j, k) and CA3-hilus area (not shown) of WNV_KUN35911_- than WNV_IS98_-infected mice. In summary, infection by the WNV_IS98_ strain gave rise to a low level of apoptosis and light inflammation, while the WNV_KUN35911_ strain induced a higher level of apoptosis and more severe inflammation in several areas of the brain. The association between the severity of inflammation and the extent of apoptosis further suggests that inflammation could trigger apoptosis. 

## Discussion

In this study, two phylogenetically distant lineage 1 WNV strains (WNV_IS98_ and WNV_KUN35911_) were compared as regards induction of clinical disease and apoptotic and inflammatory lesions in the brain. Doses in the range of or below those naturally inoculated by mosquito bites [27,28] were used for experimental infection in mice. We showed that, upon peripheral administration, the two strains induced distinct clinical disease and partially dissimilar lesions in the brain. Furthermore, by showing that apoptotic death and inflammation were more prominent in brains of mice infected with the less virulent strain, we suggest that virulence is not directly correlated to the severity of apoptotic and inflammatory lesions in the brain. 

Following intraperitoneal inoculation of 4-week-old C57Bl/6J mice, the WNV_KUN35911_ strain, isolated in 1984 in Australia from a sick horse [23], displayed unexpectedly high virulence (LD_50_ value of 17 PFU). Indeed, WNV_KUN_ strains had been repeatedly reported to be of low pathogenicity, both in the field, with only 18 known human cases over the last 50 years and none of them fatal [29,30], and in laboratory rodents, as reported by Beasley et al. (2002) [14]. In the latter study, WNV_KUNMRM16_ and WNV_KUNK5463_ were weakly virulent and weakly neuroinvasive, only rarely causing mortality in Swiss mice at doses of up to 10^4^ PFU. Recently, however, the avirulence of WNV_KUN_ strains has been called into question with the discovery of a new Australian strain, WNV_NSW2011_, closely related to WNV_KUN,_ but nevertheless responsible for an unprecedented outbreak of encephalitis among horses in New South Wales in 2011 [31]. The newly discovered WNV_NSW2011_ strain exhibited two classical markers associated with WNV virulence, the glycosylation tripeptide (N-Y-S) at residues E154–156, which supports N-linked glycosylation at a conserved site on the E protein of virulent strains, and the phenylalanine residue at amino acid 653 in the NS5 protein [32,33]. In our study, partial sequencing of the viral envelope (E) and NS5 genes, common targets for WNV phylogeny [34,35], confirmed the classification of WNV_KUN35911_ within the clade 1b/Kunjin cluster but also demonstrated that virulence could not be linked to these two markers, as gene sequencing showed them to be absent (our supplementary data). Whether the genome harbours other polymorphisms suspected to be associated with virulence, such as in the NS2A, NS3, NS4B genes [36–39] or the 3’ untranslated region (UTR) [40], or other virulence determinants as yet unknown, remains to be determined. Sequencing of the full-length genome will be necessary to address this issue and may lead to the definition of new markers of virulence. Another possible explanation for the unexpected virulence of the WNV_KUN35911_ strain is that C57Bl/6J mice do not allow discrimination between strains of high and low pathogenicity, as opposed to Swiss mice in which virulence experiments are classically performed. This interpretation, however, is not supported by the study of Daffis et al. (2011) [41], in which a WNV_KUN_ strain was shown to be clearly attenuated with respect to WNV_NY99_ in C57Bl/6J mice. Thus, our study has likely revealed a second highly pathogenic WNV_KUN_ strain isolated in the New South Wales area of Australia. The high virulence of WNV_IS98_ observed in our study was more expected. WNV_IS98_ was isolated from a stork during the 1998 outbreak in Israel [22] and is closely related to the highly pathogenic WNV_NY99_ strain [42]. Consistent with a previous study showing that WNV_IS98_ and WNV_NY99_ had comparable i.p. LD_50_ values of below 10 PFU [24], the i.p. LD_50_ value of WNV_IS98_ was only 1.5 PFU in our study. Thus the LD_50_ value of WNV_IS98_ is a log lower than that observed for WNV_KUN35911_, demonstrating that it is substantially more virulent than WNV_KUN35911_ in our animal model.

We observed that both viral strains were capable of inducing encephalitis and causing death when inoculated intraperitoneally at similar doses. While in both cases, lesions were disseminated throughout the entire parenchyma, clear quantitative and qualitative differences were observed in brains infected by the 2 strains. Whereas apoptotic death was prominent in brains infected with WNV_KUN35911_, it was rare in those infected with WNV_IS98_. In pyramidal neurons of the hippocampus, apoptosis was actually undetectable in the latter case. Similar observations were made upon analysis of four neuro-inflammatory markers, which were much more prominent in WNV_KUN35911_- than in WNV_IS98_-infected mice in several brain areas. Further evidence for the distinct behaviour of the two viral strains in the brain came from observation based on cleaved caspase 3 staining. Both caspase-3-dependent and -independent apoptotic mechanisms had been previously observed by Samuel et al. (2007) [25] in brains of mice infected with WNV_NY99_. Our results further strongly suggested that different mechanisms were involved when apoptosis was induced in a particular brain area by two different strains, WNV_IS98_ inducing mainly a caspase 3-dependent apoptosis in cortex and striatum whereas it was predominantly independent of caspase-3 upon infection by WNV_KUN35911_. It is worthy of note that two WNV strains have recently been shown to behave differently in astrocytes culture. WNV_MAD78_, an avirulent strain, replicated and spread much more slowly in these cells than WNV_NY3356_, a highly pathogenic strain [43]. To our knowledge, this study and our own are the only reports showing that different WNV strains have distinct behaviour in neural cells. It would be interesting in the future to perform experiments in *in vitro* culture systems to determine whether some strains trigger neuronal apoptosis through non-caspase proteases such as calpain and cathepsin family proteins [44,45], whereas others preferentially induce caspase-dependent mechanisms. 

Several hypotheses may be advanced to explain the differences observed after inoculation of WNV_IS98_ and WNV_KUN35911_ in brain pathology and clinical disease. First, the two viral strains may have had differential capacities to enter or to replicate in the brain, reflecting a dose effect. Such dose effect has previously been shown by others to affect brain pathology [46]. However, the quantification of the virus in three brain areas did not support this hypothesis, as similar viral loads were found for both strains. Second, the viral strains may have exhibited different tropism. This is indeed the case since viral antigen was strongly expressed in the CA1 area of the hippocampus of WNV_KUN35911_- but not in WNV_IS98_-infected brains. The inverse observation was made in the dentate gyrus, with WNV_IS98_ but not WNV_KUN35911_ antigen evidenced. Interestingly, in the CA1 area of WNV_KUN35911_-infected mice, both viral antigen and apoptotic markers were strongly expressed, which is compatible with the view that the virus itself might be responsible for inducing neuronal apoptosis [25]. Like many encephalitic viruses, WNV may induce neuronal death either in a direct or indirect manner and, notably, in the latter case, through inflammation [47]. In cortex and striatum, despite similar tropism of the two viral strains, a higher level of apoptosis induced by WNV_KUN35911_ was observed and was associated with a higher level of inflammation, as shown by the increase in number and size of perivascular infiltrates. Similarly, the increase in apoptosis was associated with an increase in T cell entry, another marker of inflammation, into the cortical parenchyma and CA3-hilus area of hippocampus. These observations suggested that in these areas inflammation is at least partly responsible for cell demise. How the two viral strains differentially target the neurons and activate the inflammatory response remains unclear. As the strictest precautions had been taken in experimental design (strain and age of mice, history of passage in the same VERO cells, titration protocols, etc..), the observed differences may be due to genetic variation between the two strain. Mutations in genes involved in target recognition, such as the envelope, or in virus replication, are likely candidates, as they have been previously shown to modify organ tropism [48]. Other mutations that lead to differential regulation of cytokines and chemokines, such as type I IFN, TNFα, and CXCL10, which are involved in attraction and entry of inflammatory cells in the brain [26,49], or of the matrix metalloproteinase MMP9, involved in blood brain barrier permeability and entry of immune T cells [50], could also be implicated. In this regard, attenuated strains of rabies viruses were recently shown to induce higher levels of chemokines than pathogenic strains [51] and to induce entry of immune effector cells, whereas the highly pathogenic virus did not [52]. Nevertheless, it is striking that, in the course of infection by the WNV_IS98_ strain, Lucas et al. (2004) [24] previously observed clinical symptoms, neurotropism and hippocampal apoptosis that closely resemble the manifestations that we observed for the WNV_KUN35911_ strain. In the latter study, however, mice were older and inoculated doses of WNV_IS98_ were higher than those used in our study, possibly explaining the discrepancy between our observations. Thus, the phenotypic expression of viral infection in the brain is highly complex depending on multiple factors such as strain and age of mice, history of viral strains, doses and finally genetic variation of the virus. 

Damage caused to the CNS by different neurotropic viruses involves different mechanisms. Poliovirus, for example, causes paralysis by inducing apoptosis of motoneurons [53], the neural cells governing muscle contraction. Rabies virus uses a different strategy. It induces neuronal dysfunction by decreasing protein synthesis, but also protects the infected neurons from apoptosis, thereby allowing the virus to persist for a longer time in the CNS [54]. The most virulent strains appeared to induce the lowest extent of neuronal death. Similar observation was made in our study as a very weak level of apoptosis was found in brains of mice infected with the highly virulent WNV_IS98_ strain whereas the less virulent WNV_KUN35911_ strain showed a much higher level of apoptosis. These findings revealed that WNV virulence was not directly linked to neuronal apoptosis in the brain areas studied and suggested that the final outcome in WNV-induced disease might not be viewed as a consequence of neuronal apoptosis. Rather, neuronal dysfunction might be responsible for clinical disease and ultimately the death of infected animals, a phenomenon that has been previously described not only for rabies, as indicated above, but also for neurodegenerative disease of non-infectious origin [55–57]. Alternatively, we cannot exclude that the virulence of the two WNV strains investigated may be linked to apoptosis of a specific pool of neurons involved in critical functions of the body, which would not have been revealed in our study. Recently, the study by Morrey et al. (2012) [58] suggested that neurological lesions affecting respiratory function may be the primary cause of WNV-induced death. Whether dysfunction or apoptosis of neurons involved in breathing is linked to virulence remains to be elucidated. It is thus not yet clear how virulence and brain pathology are related. Further understanding of this link is necessary to promote the development of novel therapeutic targets for improving acute and long-term neurological complications of WNV-induced neuroinvasive disease. 

## Materials and Methods

### Ethics statement

This study was performed in strict accordance with the French guidelines and recommendations on animal experimentation and welfare. Experiments were carried out under the authority of licence issued by the Direction des Services Vétérinaires (Dr M Coulpier, accreditation number 94-046-34) and the specific protocol for this study was approved by the local Animal Ethics Committee (ANSES/ENVA/UPEC, permit number 15/02/11-02). Every effort was made to minimize suffering.

### Viruses

WNV infections were performed with two viral isolates, IS-98-ST1 (WNV_IS98_) and KUNJIN 35911 (WNV_KUN35911_), which were kindly provided by Dr. P. Desprès (Pasteur Institute, France). Both strains were passaged once in VERO cells (ATCC CCL-81) to generate a virus stock that was used in all experiments. Titers were determined in a standard plaque assay and expressed as plaque forming units (PFU) as previously described [59]. Briefly, 7x10^5^ VERO cells were seeded into each well of a 6-well plate and infected with WNV for 90 min at 37°C. Cells were overlaid with 2% agarose (SeaPlaque, Lonza) in MEM (Gibco) containing 5% fetal bovine serum (FBS, Lonza), 1% sodium pyruvate (Invitrogen Life Technologies), penicillin (1U/ml, Invitrogen Life Technologies) and streptomycin (1µg/ml, Invitrogen Life Technologies). Seventy-two hours after infection cells were fixed with 4% paraformaldehyde (Electron Microscopy Science) and stained with 0.4% crystal violet (Alfa Aesar) for 24 h in a humid chamber at 37°C. Lysis plaques were counted after removal of the agarose layer. 

### Mouse experiments

Three-week-old female C57Bl/6J mice were purchased from Charles River Laboratories (L’Arbresle, France). After one week of acclimatization in a biosafety level 3 animal facility, peripheral infection was performed by intraperitoneal (i.p.) inoculation of 100 µl of virus with doses ranging from 0.1 to 10^5^ PFU diluted in phosphate buffered saline (PBS, Gibco, endotoxin free, pH 7.4). PBS alone was used as a control. All mice were weighed daily and observed twice a day for clinical disease. Clinical signs included significant weight loss, weakness, ruffled fur, hunched posture, ataxia, tremors and occasionally hind leg paralysis. Mice were euthanized by lethal injection of Pentobarbital (CEVA Santé animale, France) either 15 days after infection when healthy, or at a pre-mortem stage when diseased. Four additional mice of each group inoculated either with PBS or 10^2^ PFU of the 2 WNV strains were euthanized 6 days after infection (TUNEL analyses). Brains were then immediately removed and the two hemispheres were separated and prepared either for histological analyses (right hemisphere) or RNA extraction (left hemisphere) as described in the corresponding sections. The fifty percent lethal dose (LD_50_) was calculated by the method of Reed and Muench (1938). 

### RNA extraction and quantitative RT-PCR

Half-brains were dissected into cerebral hemisphere, cerebellum and brainstem, and were soaked in RNAlater (Ambion) overnight at +4°C, before storage at -20°C. RNA was extracted using commercially available materials (RNeasy mini kit, Qiagen) according to the manufacturer’s instructions, reverse-transcribed and amplified as described by Bahuon et al. [59]. The AgPath-ID one-step RT-PCR Kit (Applied Biosystems, Carlsbad, CA) was used with a primers/probe set targeting the WNV 5’ untranslated region (5’UTR), WNproC-10F 5’-CCTGTGTGAGCTGACAAACTTAGT-3’, WNproC153R 5’- GCGTTTTAGCATATTGACAGCC-3’ and probe 5’-FAM-CCTGGTTTCTTAGACATCGAGATCT-TAMRA-3’) [60] and a primers/probe set targeting a cellular gene, β-actin, ACTB-966F 5’-CAGCACAATGAAGATCAAGATCATC-3’, ACTB-1096R 5’-CGGACTCATCGTACTCCTGCTT-3’ and probe ACTB1042-67 5’-VIC-TCGCTGCCACCTTCCAGCAGATGT-TAMRA-3’ [61]. Each sample was quantified in duplicate. Absolute quantification was performed using WNV RNA that had been transcribed *in vitro*, and β-actin RNA, kindly provided by the French FMDV Reference Laboratory (ANSES, Maisons-Alfort). 

### Histology and immunohistochemistry

Half-brains were fixed in 4% formalin (Labonord) for 4 days, post-fixed for 2 additional days followed by dehydration, clearing and embedding in paraffin wax. Four micrometer thick serial coronal sections, corresponding to section N°19 of the mouse brain library (http://www.mbl.org/) were prepared and either stained with hematoxylin-eosin-saffron (HES) or immunostained with antibodies directed against the neuronal nuclear Neu-N protein (1/500, Chemicon), the glial fibrillary acid protein (GFAP) protein (1/1000, Dako), the WNV envelope protein (E-24, 1/3000, kindly provided by Dr. P. Desprès, Pasteur Institute, France) [62,63] and the CD3 lymphocyte marker (1/200, Dako). For immunohistochemistry, antigen retrieval was achieved by heating sections in 10 mM sodium citrate buffer at pH 6 (Sigma), before saturation with 3% bovine serum albumin (BSA, Sigma) in PBS and addition of antibodies. For anti-Neu-N, anti-GFAP and anti-CD3 antibodies, sections were subsequently incubated with a species-specific biotinylated secondary antibody (1/300, Vector Laboratory) and visualized using the avidin-biotin-peroxidase method, according to the manufacturer’s instructions (ABC kit, Vector Laboratory), and the Vector-VIP solution (Vector Laboratory). For detection of WNV envelope protein, the anti-E-24 antibody was directly coupled with the Cy-3 fluorophore. No staining was observed when first antibodies were omitted in both PBS-inoculated and infected brains. 

### Quantification of inflammatory response, neurons and apoptosis

Perivascular infiltrates and meningitis were observed and quantified based on HES staining in cortex, striatum, hippocampus, cerebellum and brainstem. Three sections per animal were analyzed at 200x magnification (Zeiss Imager Z1, Axio Cam HRH, AxioVision 4.6.3) by two different experimenters. Classification of perivascular infiltrates was carried out as follows: grade 1 corresponded to infiltrates with 1 row of immune cells, grade 2 of 2 rows of inflammatory cells and grade 3 of 3 or more rows. Similarly, meningitis was classified in three distinct categories according to the number of rows of lymphocytes in the meninges. Mild meningitis was characterized by 1 row of lymphocytes, moderate meningitis by 2 rows and severe meningitis by 3 or more rows.

On the basis of anti-Neu-N immunostaining, pyramidal neurons of the CA1 area in hippocampus were enumerated in two sections per animal (220µm field) under light microscopy at 200x magnification (Zeiss Imager Z1, Axio Cam HRH, AxioVision 4.6.3). 

Apoptosis was quantified using 2 apoptotic markers, activated caspase 3 and the DeadEnd Fluorometric TUNEL system (Promega). Sections were first immunostained with cleaved caspase 3 antibody (1/200, Cell Signaling) followed by Alexa Fluor 555-labelled anti-rabbit immunoglobulin (1/1000, Invitrogen), and then processed according to the manufacturer’s instructions for TUNEL staining. Nuclei were counterstained with DAPI. TUNEL- and cleaved caspase 3-positive cells as well as DAPI-positive nuclei were enumerated in 2 independent fields of 0.15 mm^2^ in cortex, striatum, cerebellum and brainstem, and in one field in the hippocampus. Two sections per mouse were counted. Quantification was performed under fluorescent microscopy at a 200x magnification (Zeiss ApoTome, HXP 120 camera, AxioVision 4.6.3). 

### Statistical analyses

Results are expressed as mean ± standard deviation (SD). Statistical comparisons between groups were performed using Wilcoxon and one-way ANOVA tests. Graphs were made using GraphPad Prism version 4.03 (GraphPad Software, San Diego, California, USA).

## Supporting Information

Figure S1 **WNV_KUN35911_ belongs to clade 1b and is devoid of 2 known virulence markers.** The E (nt 1363-1643 of WNV GenBank accession no. FJ51394.1) and NS5 (nt 9040-10124) genes of WNV_IS98_ and WNV_KUN35911_ were sequenced (Eurofins MWG Operon) and their deduced amino acid sequences aligned using the ClustalW software (VectorNTI Advance 11). (A) Phylogenetic tree of WNV strains based on a 671nt fragment of the NS5 gene (Genbank accession number JX014270.1). It was constructed with the program MEGA (v5.01) by using the neighbour-joining algorithm. Bootstrap confidence level (1000 replicates) and a confidence probability value based on the standard error test were calculated using MEGA. According to the convention for naming WNV strains, a set of letters indicates the place where the strain was isolated (Ro=Romania, Rus=Russia, Fr=France, It=Italy, NY=New York, Is=Israel, Ind=India, SA=South Africa, Ug=Uganda, Hu=Hungary, Gr=Greece, Sp=Spain, Rab=Rabensburg,) and a 2 digit number indicates the isolation year (e.g., 00=2000, 96=1996) and GenBank accession number. JEV, a closely related flavivirus, was used to root the phylogenetic tree. (B) Alignment of E protein, amino acids 428-513. The N-glycosylation site is indicated by an asterisk. Note that it is lacking in WNV_KUN35911_ (C) Alignment of NS5 protein, amino acids 3142-3249. The phenyalanine aa is indicated by an asterisk. Note that it has been replaced by a serine in WNV_KUN35911_. Identical residues appear in yellow, while different residues are shown in white.
(DOC)
