# Supplementary material for: Comparison of the Neuropathology Induced by Two West Nile Virus Strains
Source: PLoS One. 2013 Dec 18;8(12):e84473. doi: 10.1371/journal.pone.0084473 (PMC3867487; doi:10.1371/journal.pone.0084473)
Supplement: Figure S1 — WNVKUN35911 belongs to clade 1b and is devoid of 2 known virulence markers. The E (nt 1363-1643 of WNV GenBank accession no. FJ51394.1) and NS5 (nt 9040-10124) genes of WNVIS98 and WNVKUN35911 were sequenced (Eurofins MWG Operon) and their deduced amino acid sequences aligned using the ClustalW software (VectorNTI Advance 11). (A) Phylogenetic tree of WNV strains based on a 671nt fragment of the NS5 gene (Genbank accession number JX014270.1). It was constructed with the program MEGA (v5.01) by using the neighbour-joining algorithm. Bootstrap confidence level (1000 replicates) and a confidence probability value based on the standard error test were calculated using MEGA. According to the convention for naming WNV strains, a set of letters indicates the place where the strain was isolated (Ro=Romania, Rus=Russia, Fr=France, It=Italy, NY=New York, Is=Israel, Ind=India, SA=South Africa, Ug=Uganda, Hu=Hungary, Gr=Greece, Sp=Spain, Rab=Rabensburg,) and a 2 digit number indicates the isolation year (e.g., 00=2000, 96=1996) and GenBank accession number. JEV, a closely related flavivirus, was used to root the phylogenetic tree. (B) Alignment of E protein, amino acids 428-513. The N-glycosylation site is indicated by an asterisk. Note that it is lacking in WNVKUN35911 (C) Alignment of NS5 protein, amino acids 3142-3249. The phenyalanine aa is indicated by an asterisk. Note that it has been replaced by a serine in WNVKUN35911. Identical residues appear in yellow, while different residues are shown in white. (DOC) [file pone.0084473.s001.doc]

**Figure S1**

A)

Clade 1b

Lineage 2

Lineage 3

Lineage 4

Lineage 1

Clade 1a

Lineage 5

Putative lineage 8

B)


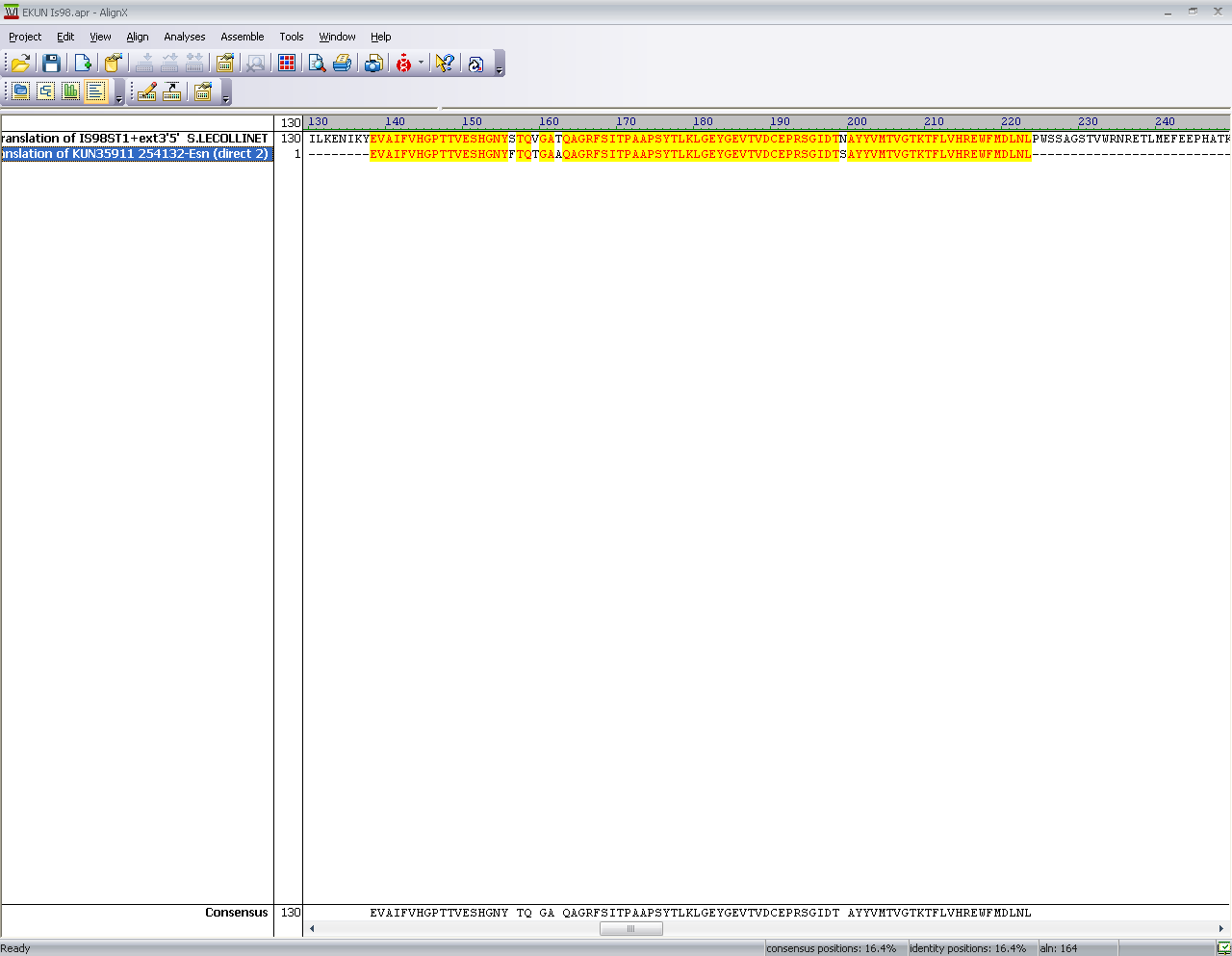

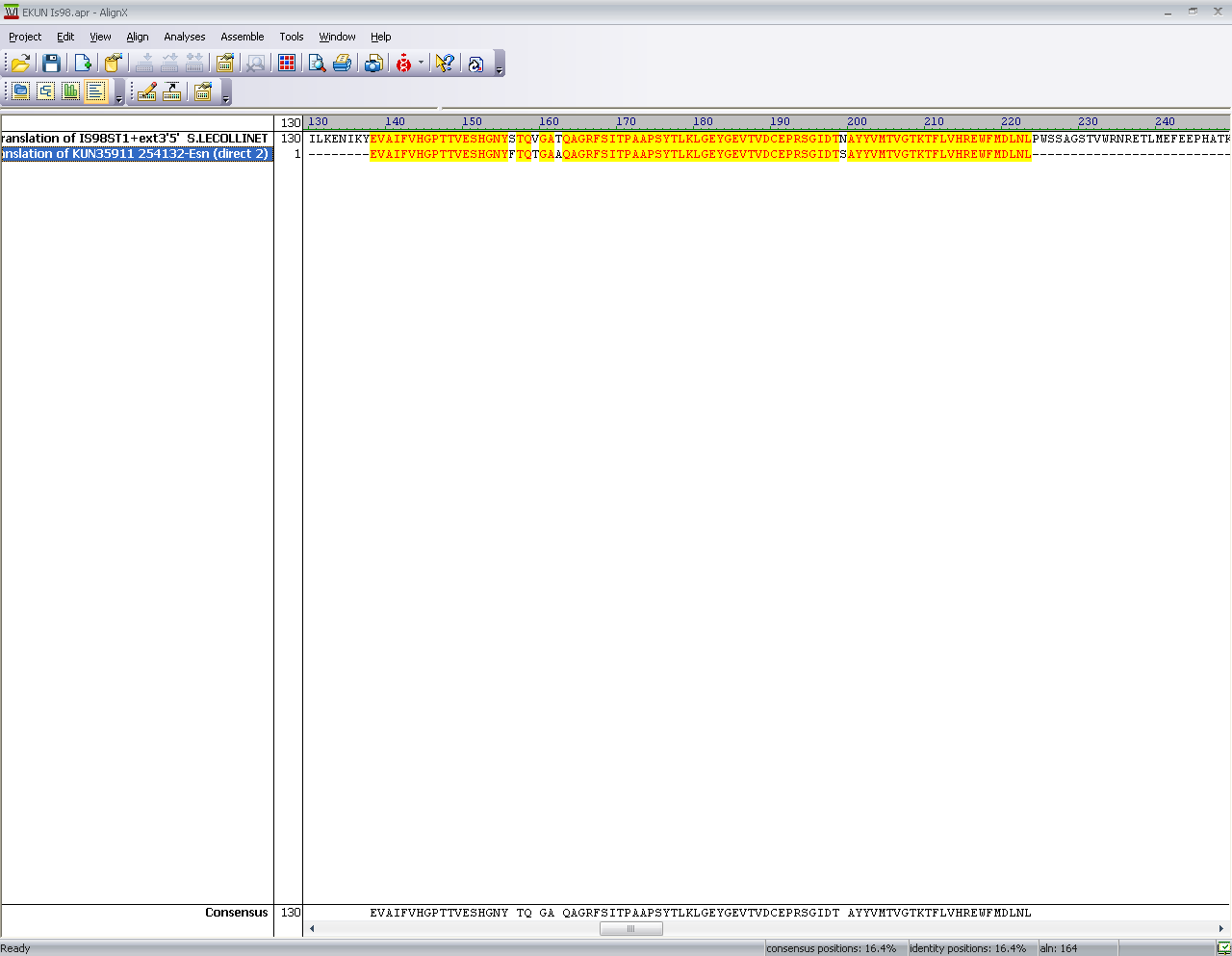


🞹

WNV_IS98_ :

WNV_KUN35911_ :

Consensus :

C)


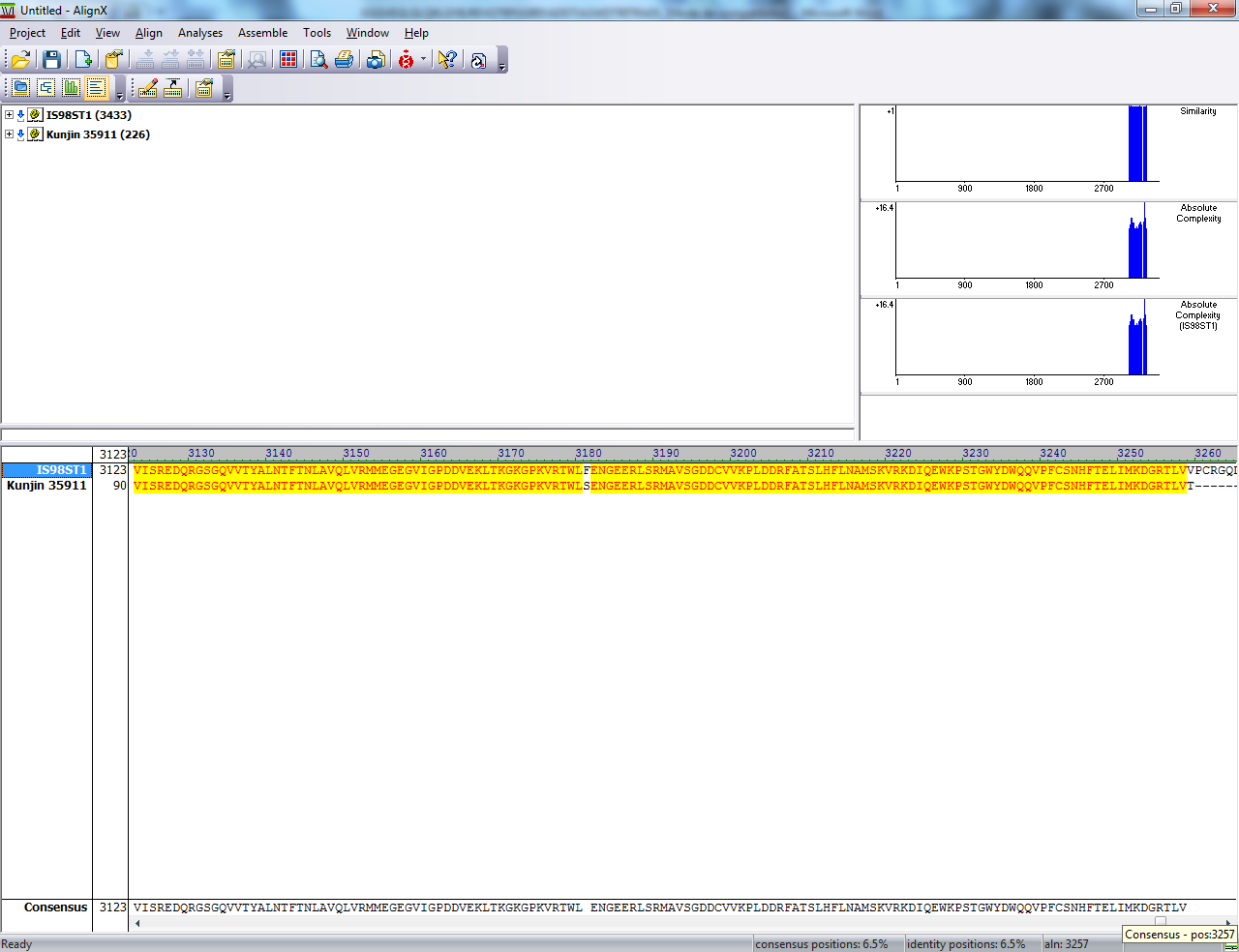

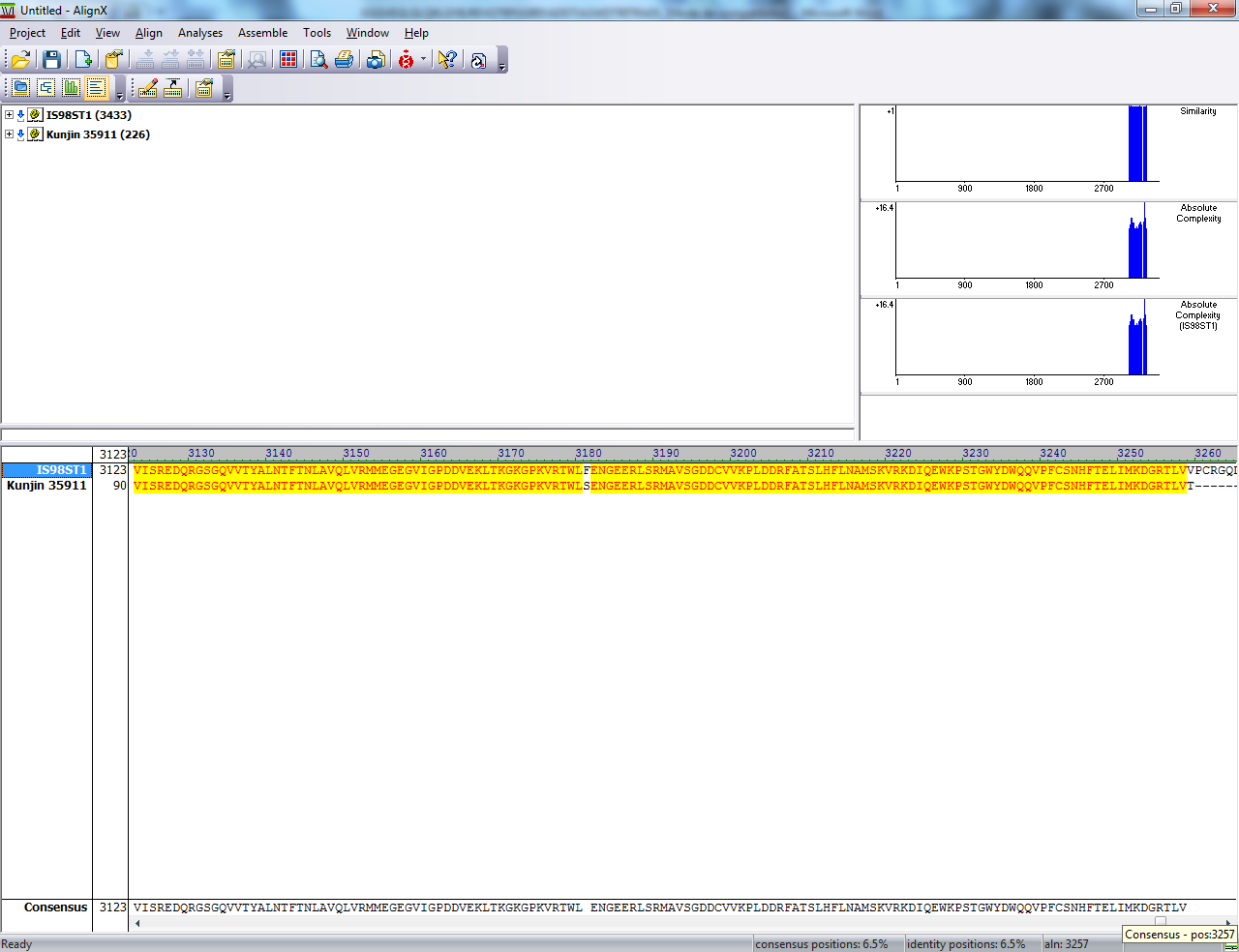


🞹

WNV_IS98_ :

WNV_KUN35911_ :

Consensus :
